# Overexpression of the human heat shock protein B1 alters obesity-related metabolic changes in a sex-dependent manner in a mouse model of metabolic syndrome

**DOI:** 10.1186/s13293-025-00746-z

**Published:** 2025-08-25

**Authors:** Zsófia Ruppert, Márta Sárközy, Bettina Rákóczi, Brigitta Dukay, Petra Hajdu, Gergő Szűcs, Zsolt Galla, Ákos Hunya, Ferenc Kovács, András Kriston, Péter Monostori, Péter Horváth, Gábor Cserni, László Tiszlavicz, Tamás Csont, László Vígh, Miklós Sántha, Zsolt Török, Melinda E. Tóth

**Affiliations:** 1 Laboratory of Molecular Stress Biology, Institute of Biochemistry, HUN-REN Biological Research Centre, Szeged, Hungary; 2 Faculty of Science and Informatics, Doctoral School in Biology, University of Szeged, Szeged, Hungary; 3 Department of Pathophysiology, Albert Szent-Györgyi Medical School, University of Szeged, Szeged, Hungary; 4 MEDICS Research Group, Department of Biochemistry, Albert Szent-Györgyi Medical School, University of Szeged, Szeged, Hungary; 5 Metabolic and Newborn Screening Laboratory, Department of Pediatrics, Albert Szent-Györgyi Medical School, University of Szeged, Szeged, Hungary; 6 Synthetic and Systems Biology Unit, Institute of Biochemistry, HUN-REN Biological Research Centre, Szeged, Hungary; 7 Single-Cell Technologies Ltd, Szeged, Hungary; 8 Institute of AI for Health, Helmholtz Zentrum München, Neuherberg, Germany; 9 Department of Pathology, Albert Szent-Györgyi Medical School, University of Szeged, Szeged, Hungary

**Keywords:** Metabolic syndrome, Apolipoprotein B-100, High-fat diet, Heat-shock protein, HSPB1, Sex-dependency

## Abstract

**Background:**

Obesity is a global health challenge that can lead to various complications, such as metabolic syndrome, diabetes mellitus, and cardiovascular diseases. Heat shock proteins are evolutionarily conserved chaperones that help maintain cellular protein homeostasis. Their expression is dysregulated in various chronic diseases, including diabetes mellitus and hyperlipidemia, and they also regulate inflammatory processes. Therefore, the present study aimed to investigate the effects of a small heat shock protein, HSPB1, on the comorbidities and complications of obesity in a transgenic mouse model.

**Methods:**

Male and female human apolipoprotein B-100 (APOB) transgenic mice fed with a high-fat diet (HFD) from months 3–10 of age were used as a model of metabolic syndrome (MetS). To study whether HSPB1 influences the development of MetS, APOB animals were crossed with HSPB1-overexpressing mice. Age and sex-matched wild-type and human HSPB1-overexpressing mice were used as controls. Changes in cardiac morphology and function were assessed by transthoracic echocardiography at month 9. At month 10, serum triglyceride and cholesterol concentrations were determined by enzymatic colorimetric assays. Pathological changes in the liver were studied on hematoxylin–eosin-stained sections. Expression levels of genes involved in inflammation and metabolism were measured by quantitative real-time polymerase chain reaction in the liver, left ventricle, and visceral white adipose tissue (vWAT).

**Results:**

The body weight and serum LDL-cholesterol levels were significantly higher in the APOB animals than in the wild-type mice in both sexes. Notably, HSPB1 overexpression further increased weight gain in female APOB animals. Conversely, in APOB males, HSPB1 overexpression decreased LDL-cholesterol levels without significantly affecting body weight. Furthermore, in APOB females, HSPB1 overexpression elevated *Fgf-21* expression in the vWAT, restored *Lpl* levels, and reduced the expression of several cytokines in the liver. APOB males developed left ventricular hypertrophy (LVH) with diastolic dysfunction. HSPB1 overexpression induced LVH without cardiac dysfunction in the wild-type animals.

**Conclusions:**

Both sexes of APOB animals developed MetS. APOB males presented LVH with preserved ejection fraction (EF); however, APOB females showed enlarged left ventricular end-systolic volume (LVESV). In APOB animals, HSPB1 overexpression exerted a sex-dependent influence on obesity-related alterations, including weight gain, hypercholesterolemia, and hepatic and vWAT gene expression.

**Supplementary Information:**

The online version contains supplementary material available at 10.1186/s13293-025-00746-z.

## Highlights

• Overexpression of human HSPB1 led to further weight gain in HFD-fed APOB-100 females, while the body weights of HFD-fed APOB-100 males were unaffected by human HSPB1.

• Human HSPB1 overexpression significantly decreased the LDL-cholesterol levels in HFD-fed APOB-100 males.

• mRNA level of fibroblast growth factor 21 was elevated in the vWAT of HFD-fed APOB-100 females in response to human HSPB1 overexpression.

• Increased gene expression level of lipoprotein lipase was restored by human HSPB1 overexpression in the liver of HFD-fed APOB-100 females.

• Overexpression of human HSPB1 failed to restore hyperlipidemia-related cardiac morphological alterations found in HFD-fed APOB-100 males, while it induced LVH in the wild-type animals of both sexes.

**Supplementary Information:**

The online version contains supplementary material available at 10.1186/s13293-025-00746-z.

## Abstract (plain language summary)

Representing the primary risk factor for several chronic diseases, including non-alcoholic fatty liver disease, type 2 diabetes mellitus, and cardiovascular diseases, obesity is a global health challenge nowadays. Cells defend themselves against various stress and disease conditions by inducing a stress response characterized by the activation of heat shock proteins. However, the levels of these proteins and the inducibility of the cellular stress response are altered in chronic metabolic diseases, such as diabetes or hyperlipidemia. Here, we aimed to analyze whether a small molecular weight heat shock protein, HSPB1, has an effect on MetS using a mouse model. We found that HSPB1 overexpression led to a further increase in weight gain and blood LDL-cholesterol concentration in female disease model animals. Conversely, in males, HSPB1 overexpression led to a decrease in LDL cholesterol levels without any significant impact on body weight. However, despite the higher body weight, none of the investigated comorbidities, such as inflammation, hepatic steatosis, or cardiac dysfunction, were worsened in the disease model females in response to HSPB1 overexpression, supported by the sex-specific alterations in the gene expression pattern in these tissues. These results suggest that HSPB1 may have a complex regulatory role in obesity-related comorbidities. Although the restoration of the heat shock response and levels of heat shock proteins may be an effective therapeutic strategy in metabolic disorders, it is important to consider sex-based differences to ensure optimal outcomes.

**Supplementary Information:**

The online version contains supplementary material available at 10.1186/s13293-025-00746-z.

## Background

The global prevalence and impact of obesity have reached epidemic proportions, showing a worsening tendency over the last five decades [1]. After smoking, obesity became the second leading cause of preventable death since it represents the primary risk factor for several chronic diseases, including non-alcoholic fatty liver disease (NAFLD), type-2 diabetes mellitus, cardiovascular diseases, and chronic kidney disease [2, 3]. The prevalence of these chronic diseases is increased by metabolic syndrome (MetS), which is a cluster of cardio-metabolic risk factors, including abdominal obesity, dyslipidemia, increased serum glucose levels, and hypertension [4, 5].

Chronic high-calorie intake increases the size of adipocytes and induces capillary rarefaction, leading to hypoxia, mitochondrial dysfunction, and the dysregulation of fatty acid homeostasis [6–9]. These factors can trigger enhanced adipokine and cytokine secretion in the adipose tissues, resulting in systemic, low-grade inflammation and dysfunctional immune response, which may aggravate various obesity-related disorders [10]. Several pro-inflammatory cytokines secreted by the adipose tissue, including interleukin-6 (IL-6), interleukin-1β (IL-1β), and tumor necrosis factor-α (TNFα), have been shown to reduce the insulin sensitivity of adipose tissue, liver, and skeletal muscle, finally leading to systemic IR in obese patients [11]. Moreover, IR is associated with altered lipid and lipoprotein metabolism, resulting in hypertriglyceridemia and decreased high-density lipoprotein (HDL) cholesterol levels, which are characteristics of MetS. At the same time, dyslipidemia may also have a mutual influence on insulin signaling, as the accumulation of triglycerides (TG) in the liver can contribute to the development of hepatic IR and NAFLD [12]. NAFLD can range from mild steatosis to more severe non-alcoholic steatohepatitis and ultimately progresses to irreversible fibrosis and cirrhosis [13]. Finally, several studies have shown that MetS can increase the risk of cardio- and cerebrovascular diseases, such as heart failure, myocardial infarction, and stroke [14–17]. In addition, decreased serum HDL or impaired fasting glucose levels are associated with a poor prognosis for patients with cardiovascular diseases, increasing their all-cause mortality risk [18].

Expression levels of the heat shock proteins (HSPs) are altered in various chronic diseases, including metabolic disorders [19]. The main function of these evolutionarily conserved *chaperone* proteins is to maintain protein homeostasis, illustrating their protective roles against protein misfolding [19, 20]. Moreover, several moonlighting functions of HSPs, ranging from membrane protection to immunomodulation, have been revealed in the last decades [21–25]. Inducing HSPs by mild stress pretreatment (precondition) was protective against acute injuries, including cardiac ischemic stress [26]. However, the levels of HSPs and the inducibility of the heat shock response are altered in chronic metabolic diseases, such as diabetes mellitus or hyperlipidemia [27, 28]. Therefore, restoring the heat shock response and levels of HSPs may be an effective therapeutic strategy in metabolic disorders. Indeed, several studies showed that inducing cellular stress response by physical exercise or hot tub treatment can reduce IR, inflammatory cytokine release, and even body weight [29, 30]. Moreover, heat treatment reduced serum TG and LDL-cholesterol levels and increased HDL-cholesterol concentrations in LDL receptor knockout mice [31]. Heat therapy or exercise activates the heat shock response, in which HSPs form a complex cooperating network. Therefore, it is challenging to determine the precise role of each participant in the process. Most studies focus on the role and clinical relevance of HSP70. Its concentration is differently affected by diabetes mellitus in different tissues [32]. Increasing the level of HSP70 through transgenic overexpression, hyperthermia, or pharmacological induction (e.g., BGP15) was also shown to prevent hyperglycemia, hyperinsulinemia, and IR in different diabetes mellitus or obesity models [19, 33, 34]. However, the role of the small molecular weight HSPB family in metabolic disorders is not yet fully understood [35]. In addition to their intracellular chaperone role, emerging evidence suggests that HSPs may also have extracellular functions. Previously, HSPB5, a member of the small HSP family, was proposed to act as an adipokine, as it was found to be secreted by adipocytes and presented in higher concentrations in the blood of obese patients [36]. Moreover, HSPB5 and HSPB2 have been suggested to contribute to the development of diet-induced obesity-related disorders, as these alterations failed to appear in HSPB5/HSPB2 knockout mice [37]. Accordingly, we recently found an increase in HSPB5 (*Cryab*) gene expression in the vWAT of high-fat/high-fructose-treated mice, showing a strong correlation with the expression level of leptin (*Lep*) [38], further suggesting its potential role in adipose tissue function and systemic metabolism. Moreover, small HSPs, including HSPB1, are also involved in the regulation of inflammation by acting as chaperokines [22, 23]. As chronic low-grade systemic inflammation is central to developing obesity-related metabolic dysfunction, small HSPs may have an important role in these conditions by fine-tuning the inflammatory processes and influencing metabolic functions.

In our present study, we aimed to study whether HSPB1 overexpression influences the development of MetS. We used our previously characterized mouse model, a high-fat diet (HFD)-fed apolipoprotein B-100 (APOB-100)-overexpressing transgenic mouse strain. In this obesity and MetS model, APOB-100 overexpression leads to an increased low-density lipoprotein (LDL)/HDL ratio and a more human-like serum lipid profile in mice [39, 40]. Beyond obesity and dyslipidemia, further characteristics of MetS, including elevated fasting glucose levels, systemic inflammation, NAFLD, and mild cardiovascular dysfunction, can also be observed in this APOB-100-overexpressing mouse model; however, in a sex-dependent manner [41, 42]. We crossed APOB-100- and HSPB1-overexpressing mice fed with an HFD from month 3 to month 10 of age. At the endpoint, we analyzed weight gain, serum glucose, TG and cholesterol levels, hepatic lipid accumulation, gene expression of inflammatory cytokines in the adipose tissue, and cardiac morphology and functional changes. Male and female animals were studied in separate groups due to the previously observed sex differences in the APOB-100 model.

## Methods

### Animals

Experiments conformed to the EU Directive 2010/63/EU and were approved by the regional Animal Research Ethics Committee of Csongrád-Csanád County (Csongrád-Csanád County, Hungary; project license: XVI/766/2018). All institutional and national guidelines for the care and use of laboratory animals were followed.

The human APOB-100- and the human HSPB1-overexpressing mouse strains were previously established and used by our group [39, 43]. For the generation of the human APOB-100-overexpressing strain, the construct contained the complete transcription unit, including the human APOB-100 gene, the long 5’ promoter, as well as intragenic and 3’ specific enhancer/silencer sequences, resulting in a high level of transgene expression in the liver [39, 40]. To produce the human HSPB1-overexpressing strain, the transgenic DNA construct contained the human HSPB1 cDNA driven by a cytomegalovirus promoter. Expression of the transgenic protein was confirmed in several tissues, including the brain [43], cardiac tissue, and vWAT (Fig. S1). Both strains were bred and maintained hemizygous on a C57BL/6 genetic background. In order to create double transgenic mice, we mated hemizygous human APOB-100-overexpressing and hemizygous human HSPB1-overexpressing animals. To determine the genotype of the offspring mice, DNA from tail biopsies of 10-day-old pups was purified, and the presence of the transgenes was detected by PCR, as described previously [39, 43].

**Fig. 1:**
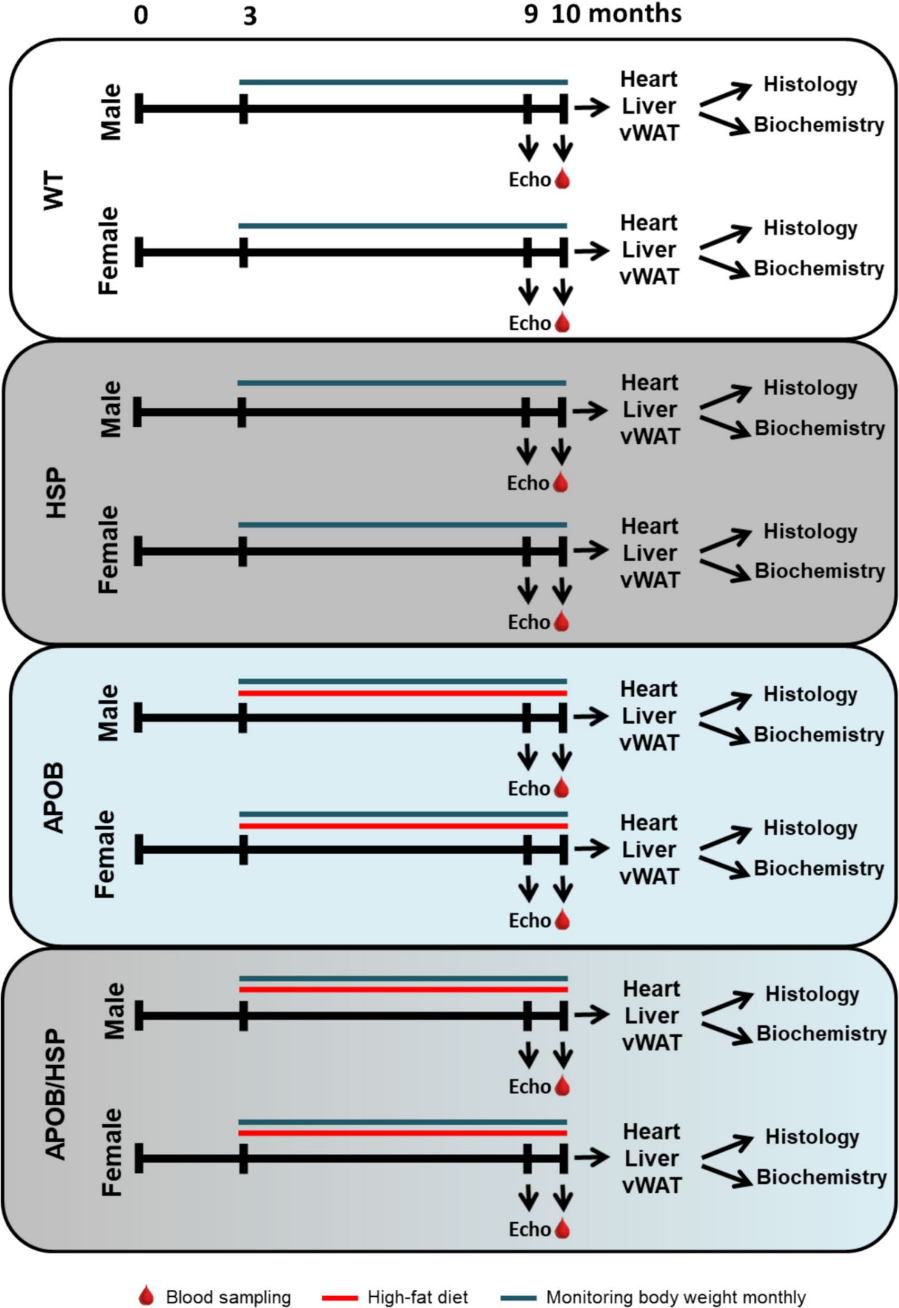
Protocol figure. Mice were divided into eight groups (n = 12). Male and female wild-type (WT) and human HSPB1-overexpressing (HSP) mice were fed a standard diet and used as controls. Sex-matched human APOB-100 (APOB)- and human APOB-100/HSPB1 (APOB/HSP)-overexpressing mice were fed with a high-fat diet to induce MetS. Dietary intervention and monthly body weight measurements started at 3 months of age and lasted 7 months. At 9 months of age, transthoracic echocardiography was performed. At 10 months of age, all mice were terminally anesthetized by sodium pentobarbital, and fasting blood samples were collected through a cardiac puncture to measure serum lipid, glucose, and uremic toxin levels. Before removing hearts, livers, and visceral white adipose tissues (vWAT), transcardial perfusion was performed. In a subgroup of animals, the subvalvular areas of the left ventricular, liver, and vWAT samples were frozen in liquid nitrogen, and then RNA was isolated. Left ventricular and hepatic samples were fixed in 4% paraformaldehyde for hematoxylin–eosin staining

In our present experiments, a total of 96 age-matched male (C57BL/6 wild-type [WT], 26–35 g; human APOB-100-overexpressing [APOB], 26–33 g; human HSPB1-overexpressing [HSP], 23–35 g; and APOB/HSP, 25–32 g) and female (WT, 21–25 g; APOB, 20–24 g; HSP, 20–28 g; and APOB/HSP, 20–28 g) mice were divided into 8 groups (n = 12) (Fig. 1). Two or three mice were housed per cages in the same room under controlled conditions (24 °C, 12–12 h light–dark cycle) throughout the experiment. Food and water were available ad libitum.

### Experimental set-up

WT mice on a standard diet (SD) were used as healthy controls. Human HSPB1 transgenic mice on SD were used to study the effects of hHSPB1 overexpression in healthy animals. APOB and APOB/HSP mice were fed an HFD (Special Diet Services, UK) to induce obesity and cardiovascular complications (Fig. 1) [41, 42]. For detailed diet composition, see Table S1. The dietary intervention started at 3 months of age and lasted 7 months (Fig. 1). Body weight was measured at baseline and monthly thereafter. At 9 months of age, transthoracic echocardiography was performed (Fig. 1). At 10 months of age, all mice were terminally anesthetized by sodium pentobarbital (*ip.* 150 µg/g). Then, fasting blood samples were collected through a cardiac puncture to measure standard laboratory parameters, including serum glucose (Accu-Chek, Roche), TG, LDL and HDL-cholesterol levels. After transcardial perfusion (with 0.9% sodium chloride in 0.01 M phosphate-buffered saline [PBS], pH = 7.4), the heart, liver, and vWAT were removed. Organ weights were measured, and samples were frozen in liquid nitrogen for RNA isolation or fixed in 4% paraformaldehyde (solved in 0.1 M PBS, pH = 7.4) for histology (Fig. 1).

### Western-blot analysis

Protein level of transgenic human HSPB1 was determined from heart, liver, and vWAT (samples were pooled from 8 to 12 animals/group). Samples were homogenized with Bullet Blender (Next Advance, Inc., Troy, NY, USA) according to the manufacturer’s instructions, in 1 mL radioimmunoprecipitation assay buffer supplemented with protease inhibitor cocktail (1 mM aminocaproic acid, 1 mM benzamidine, 1 mM phenylmethylsulfonyl fluoride). Total protein content was measured using a BCA™ Protein Assay Kit (Thermo Scientific, Waltham, MA, USA). Samples were loaded in a total concentration of 20 µg/lane and separated by a 12% sodium dodecyl sulfate polyacrylamide gel. Proteins were then blotted onto polyvinylidene difluoride membranes (Immobilon-P; Millipore, MA, USA) using a semi-dry blotting method. Membranes were then blocked with blocking buffer (0.1% Tween-20 and 5% dried skim milk in Tris-buffered saline) for 1 h at room temperature, and probed with primary antibody that recognized human HSPB1 (1:1000, ADI-SPA-803, Enzo Life Sciences, Farmingdale, NY, USA) at 4 °C overnight. Blots were then washed three times in Tris-buffered saline–0.1% Tween-20, incubated with peroxidase-conjugated anti-rabbit IgG antibody (A9169, Sigma–Aldrich, St. Louis, MO, USA) for 1 h at room temperature, diluted 1:80,000 in Tris-buffered saline–0.1% Tween-20 containing 3% dried skim milk. Subsequently, immunoreactive proteins were visualized with Immobilon Western Chemiluminescent HRP Substrate (Merck-Millipore, Burlington, MA, USA) according to the supplier’s instructions. Enhanced chemiluminescence was detected using AlphaView-FluorChem FC3 (Cell Biosciences, Santa Clara, CA, USA). For protein loading control, anti-GAPDH (G9545, Sigma-Aldrich, St. Louis, MO, USA) antibody was used.

### Transthoracic echocardiography

Cardiac morphology and function were assessed by transthoracic echocardiography at the age of 9 months, as we described previously [42, 44]. Mice were anesthetized with 2% isoflurane (Forane, AESICA, Queenborough Limited Kent, UK). Then, the chest was shaved, and the animal was placed supine on a heating pad. Two-dimensional, M-mode, Doppler, and tissue Doppler echocardiographic examinations were performed following the criteria of the American Society of Echocardiography with a Vivid IQ ultrasound system (General Electric Medical Systems) using a linear array 4.5–13 MHz transducer (GE 12L-RS probe) for morphology, and a phased array 5.0–11 MHz transducer (GE 12S-RS probe) for function. Data of 3 consecutive heart cycles were analyzed (EchoPac Dimension software; General Electric Medical Systems) by an experienced investigator in a blinded manner. The mean values of three measurements were calculated and used for statistical evaluation.

Systolic and diastolic wall thicknesses were obtained from a parasternal short-axis view at the papillary muscle level. The left ventricular diameters were measured using M-mode echocardiography from the short-axis view between the endocardial borders. Fractional shortening (FS) was used as a measure of cardiac contractility (FS = (left ventricular end-diastolic diameter [LVEDD] − left ventricular end-systolic diameter [LVESD])/LVEDD × 100). The left ventricular end-diastolic volume (LVEDV) and LVESV were calculated on four-chamber view images delineating the endocardial borders in diastole and systole. The stroke volume (SV) was calculated as the difference between LVEDV and LVESV. Cardiac output (CO) was calculated as the product of the SV and heart rate (HR). Ejection fraction (EF) was assessed using the formula (SV/LEVDV) × 100 to measure global systolic function. Diastolic function was assessed using pulse-wave Doppler across the mitral valve and tissue Doppler of the mitral annulus from the apical four-chamber view. The early mitral flow velocity (E), septal mitral annular velocity (e′), and their ratio (E/e′) provided an assessment of diastolic function.

### Serum triglyceride, LDL-cholesterol, and HDL-cholesterol levels

To study the effects of human HSPB1 overexpression on dyslipidemia, the serum TG, LDL and HDL-cholesterol levels were determined using commercially available enzymatic colorimetric assay kits (Diagnosticum Ltd., Budapest, Hungary) according to the manufacturer’s instructions. Blood samples were collected from every group (n = 10–12) through cardiac puncture under terminal anesthesia. After clot formation, samples were centrifuged at 4 °C, 1000 × g for 10 min. Then serum was removed and stored at −80 °C until use. Each serum sample was measured in triplicate. Test accuracy was monitored using standard lipid controls (Diagnosticum Ltd., Budapest, Hungary). The absorbance of the produced red/blue color product was measured at 493/596 nm, respectively, using a microplate reader (Multiskan FC, Thermo Fisher Scientific, Waltham, Massachusetts, USA). Values were used to calculate the serum TG, LDL and HDL-cholesterol concentrations, expressed in mmol/Liter.

### Serum uremic toxin levels

The levels of serum uremic toxins were measured according to previously published methodologies using ultra-high performance liquid chromatography-tandem mass spectrometry (UHPLC-MS/MS) [45, 46] MRM transition of indoxyl sulfate was 211.9/131.9 using −50 V as declustering potential and − 25 V as collision energy, retention time: 11.48 min. MRM transition of p-cresyl sulfate was 186.9/107.0 using − 50 V as declustering potential and − 26 V as collision energy, retention time: 12.50 min.

### Histological evaluation of left ventricular and liver sections

The subvalvular areas of the left ventricles and the liver samples were transversely cut in 5 μm sections, which were embedded in paraffin, fixed in formalin, and stained with hematoxylin–eosin or picrosirius red and fast green (PSFG) as described previously [47]. Histological slides were scanned with a Pannoramic Midi II scanner (3D-Histech, Budapest, Hungary), and digital images were captured at the magnification of × 10, × 40, and × 100.

On the hematoxylin–eosin-stained liver samples, we investigated the obesity-induced hepatic lipid accumulation. NAFLD Activity Score (NAS) was calculated by the sum of scores of steatosis (0–3), lobular inflammation (0–3), and hepatocyte ballooning (0–2). Moreover, the number and size of the lipid droplets were analyzed using the ImageJ software.

On the left ventricular samples, cardiomyocyte cross-sectional areas were measured to investigate the effects of human HSPB1 overexpression on cardiac morphology at the cellular level. The Biology Image Analysis Software (BIAS, Single-Cell Technologies Ltd., Szeged, Hungary) was used to evaluate hematoxylin-eosin-stained slides. Image pre-processing was followed by deep learning-based cytoplasm segmentation. User-selected objects were forwarded to the feature extraction module, which is configurable to extract properties from the selected cell components. Cardiomyocyte cross-sectional areas were measured by the software in 100 selected, longitudinally oriented, mononucleated cardiomyocytes on digital images from a single left ventricular transverse slide.

Cardiac fibrosis was assessed on PSFG slides with an in-house developed program as described previously [42, 45]. Briefly, this program determines the proportion of red pixels in left ventricular sections using two simple color filters. For each red–green–blue (RGB) pixel, the program calculates the color of the pixel in the hue-saturation-luminance color space. The first filter is used to detect red portions of the image. The second filter excludes any white (empty) or light grey (residual dirt on the slide) pixels from further processing using a simple RGB threshold. In this way, the program groups each pixel into two sets: pixels considered red, and pixels considered green but not red, white, or grey. Red pixels in the first set correspond with connective tissue and fibrosis. Green pixels in the second set correspond to the cardiac muscle. Dividing the number of elements in the first set by the number of elements in both sets gives the proportion of the connective tissue compartment of the heart area examined.

### RNA isolation and quantitative real-time polymerase chain reaction (qPCR)

To analyze the effects of human HSPB1 overexpression on the gene expression profile in different tissues, 6 animals were randomly selected in each experimental group. Then, total RNA was isolated from the liver, vWAT, and left ventricular samples using RNeasy Fibrous Tissue Mini Kit (Qiagen, Hilden, Germany). High Capacity cDNA Reverse Transcription Kit (Thermo Fisher Scientific, Waltham, Massachusetts, USA) was used to transcribe RNA to cDNA. Each reaction mixture contained 1 µg RNA (15 µL), 1.5 µL MultiScribe Reverse Transcriptase, 3 µL primer, 1.2 µL dNTP, 3 µL buffer, 6.3 µL RNase-free water. Parameters for the reverse transcription program were the following: incubation at 25 °C for 10 min, reverse transcription at 37 °C for 2 h, and inactivation at 85 °C for 5 min (using BioRad T100 Thermal Cycler, Hercules, California, USA). The cDNA product was finally diluted to 1:20 and used as a qPCR reaction template. For the qPCR reaction, 9 µL cDNA, 1 µL (250 nM final) primer mix (forward + reverse), and 10 µL Power SYBR Green PCR Master Mix 2x (Thermo Fisher Scientific, Waltham, Massachusetts, USA) were mixed in a total volume of 20 µL. The reaction was performed on a RotorGene 3000 instrument (Qiagen, Hilden, Germany) with the following settings: heat activation at 95 °C for 10 min, followed by 40 cycles of denaturation at 95 °C for 15 s, annealing at 60 °C for 60 s. Melting curve analysis was performed between 50 and 95 °C to verify the specificity of the amplification. Primer sequences used in qPCR reactions are listed in Table S2. The mouse glyceraldehyde-3-phosphate dehydrogenase (*Gapdh*) gene served as an internal control for normalization. Relative gene expression levels were calculated using the ^ΔΔ^Ct method.

### Statistical analysis

Statistical analysis was performed using Sigmaplot 12.0 for Windows (Systat Software Inc., San Jose, California, USA). The level of statistical significance was set at p < 0.05. All values are presented as mean ± SEM. The normal distribution of the data was checked using the Shapiro–Wilk normality test. In the case of normal distribution, two-way analysis of variance (ANOVA), followed by the Bonferroni post hoc test, was performed separately within the female and male groups. In those cases where the normality test failed, the Kruskal–Wallis test by ranks (i.e., ANOVA on ranks) was performed. In case of significant differences between groups, Dunn’s post hoc test was used after ANOVA on ranks. Due to the opposite changes in several groups, the effect of sex was also tested using the Student’s t-test (in case of normal distribution) or Mann–Whitney U test (if the normality test failed). qPCR data are presented as percent (%) of the corresponding control group. In the case of gene expression changes, normal distribution was checked using the Shapiro–Wilk normality test. In the case of normal distribution, the parametric Student’s t-test was performed for pairwise comparisons. If the normal distribution test failed, the non-parametric Mann–Whitney U-test was used for pairwise comparisons.

## Results

### Human HSPB1 overexpression leads to further weight gain in HFD-fed APOB-100 females

**Fig. 2:**
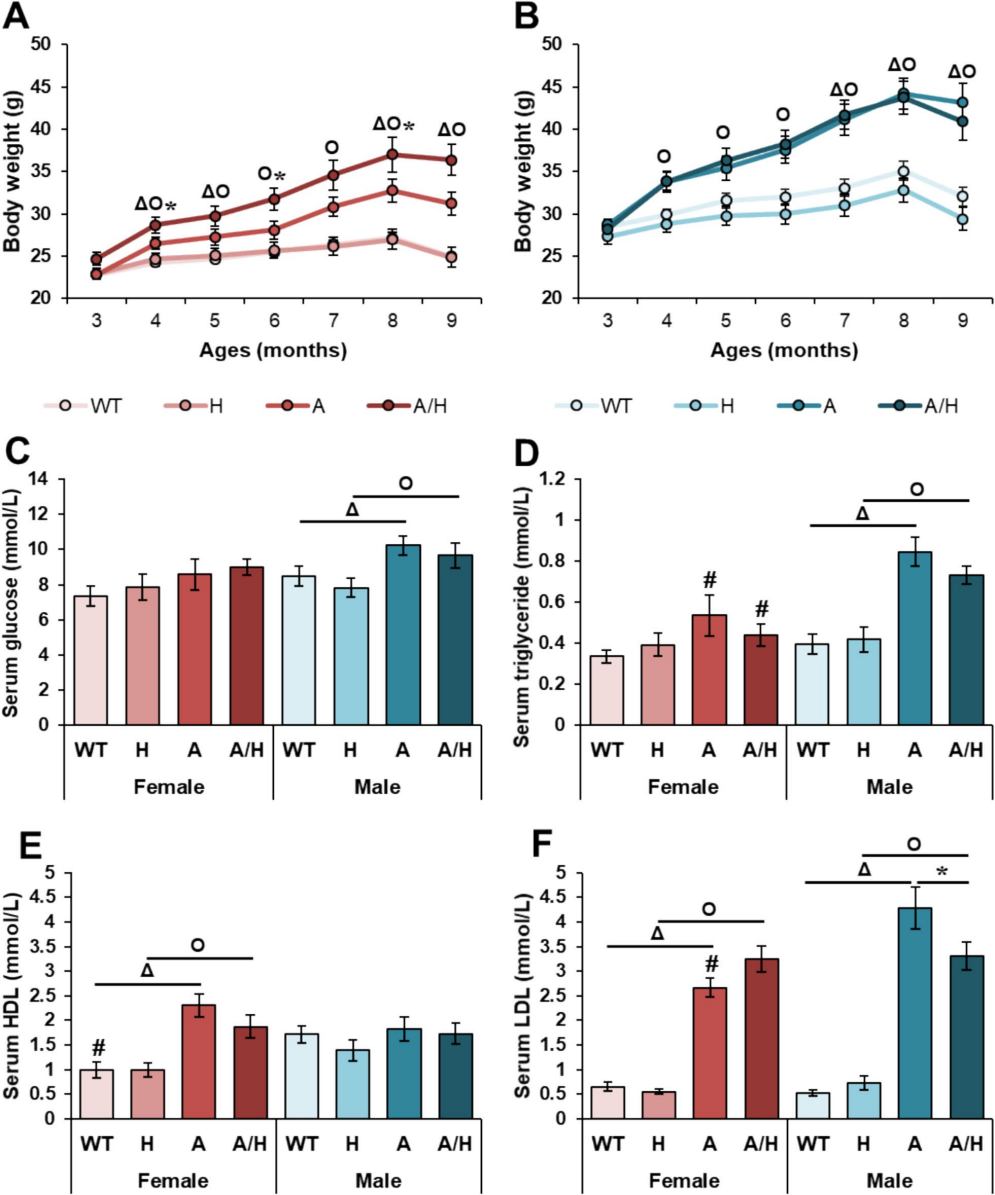
The effects of sex, MetS, and human HSPB1-overexpression on body weight and serum laboratory parameters at 10 months of age in female and male mice. Wild-type (WT) and HSPB1 (H) groups were on a standard diet. APOB-100 (A)- and APOB-100/HSPB1 (A/H) MetS model mice were fed a high-fat diet. Body weight changes of the **A** females and **B** males. Serum **C** glucose, **D** triglyceride, **E** HDL-cholesterol, and **F** LDL-cholesterol concentrations. Values are mean ± SEM; n = 11–12; ^Δ^p < 0.05, A vs. WT; ^O^p < 0.05, A/H vs H; *p < 0.05, A/H vs. A; ^#^p < 0.05 Female vs. Male

In order to characterize the weight gain of the animals from 3 months of age, the body weight of the animals was measured monthly during the 7-month-long feeding period (Fig. 1). The body weight of the APOB transgenic mice showed a consistent increase in a more pronounced manner compared to the WT controls, both in female and male groups (Figs. 2A and B). Human HSPB1 overexpression did not affect body weight in the WT groups or the APOB males. In contrast, the human HSPB1 overexpression led to further weight gain in the APOB females, as the body weights of the APOB/HSP females were significantly higher than those of the APOB females.

### Human HSPB1 overexpression decreased the LDL-cholesterol level in HFD-fed APOB-100 males

To characterize the development of IR and hyperlipidemia in the human HSPB1-overexpressing HFD-fed APOB mice, fasting serum glucose, TG, LDL and HDL-cholesterol levels were measured. In the male APOB and APOB/HSP groups, the fasting serum glucose and TG levels were significantly increased compared to the male WT and HSP groups, respectively. (Fig. 2C and D). In contrast, serum HDL-cholesterol levels were markedly elevated only in the female APOB and APOB/HSP groups compared to the female WT and HSP groups, respectively (Fig. 2E). LDL-cholesterol levels were increased in both sexes in the APOB and APOB/HSP groups compared to the sex-matched WT and HSP groups, respectively (Fig. 2F). Human HSPB1 overexpression did not affect fasting glucose, TG, or HDL-cholesterol levels in any group (Figs. 2C–E). However, the LDL-cholesterol level was significantly decreased only in the male APOB/HSP group compared to the sex-matched APOB group (Fig. 2F). In contrast, serum LDL showed an increasing trend in the female APOB/HSP group compared to the female APOB group (Fig. 2F).

### Human HSPB1 overexpression led to increased Fgf21 mRNA levels in the visceral white adipose tissue of APOB-100 females

**Fig. 3:**
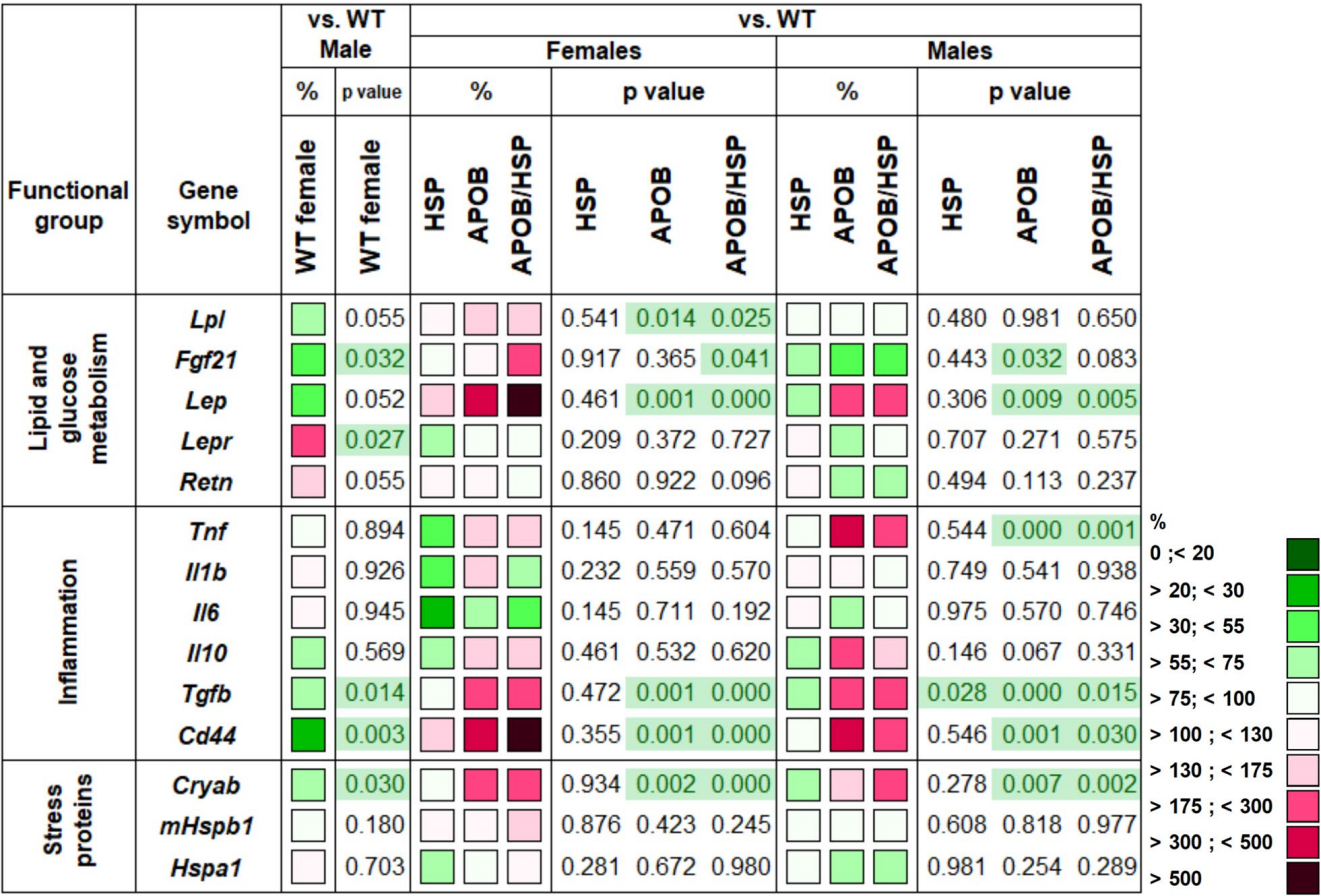
Heatmap of relative gene expression differences in visceral white adipose tissue (vWAT) in response to sex, MetS, and human HSPB1-overexpression. Relative expressions of several genes related to lipid and glucose metabolism, inflammation, and stress response were studied in the vWAT of female and male mice using qPCR (n = 6). Wild-type (WT) and HSPB1 (HSP) groups were on a standard diet. APOB-100 (APOB) and APOB-100/HSPB1 (APOB/HSP) MetS model mice were fed a high-fat diet. For the WT female vs. WT male comparison, the relative expression of target genes in females was compared to the expression levels detected in males (results are given in percentages, where the male groups’ value = 100%). For HSP vs. WT, APOB vs. WT, and APOB/HSP vs. WT comparisons, the relative expression of target genes in transgenic animals was compared to the expression levels detected in WT female and male animals separately (results are given as a percentage, where WT groups’ value = 100%)

To analyze whether sex, hyperlipidemia, or human HSPB1 overexpression influence the expression of genes involved in the regulation of inflammation, metabolism, and stress response, we isolated total RNA from the vWAT, and gene expression levels were measured by qPCR (Fig. 3). First, we compared the female and male WT groups, considering the values for the males to be 100%. The genes encoding fibroblast growth factor 21 (*Fgf21*) and the hyaluronate receptor *(Cd44)* showed remarkably lower levels in the vWAT of WT females compared to the males (42%, p < 0.05 and 27%, p < 0.01, respectively). In contrast, the mRNA level of the leptin receptor (*Lepr*) was significantly higher in females than in males (191%, p < 0.05).

Then we analyzed the differences in gene expression patterns between the WT and transgenic animals, comparing the values for the HSP, APOB, or APOB/HSP groups to the WT groups, separately in female and male animals, considering the values for the WT groups as 100%. While the expression level of the lipoprotein lipase (*Lpl*) was not influenced by the transgenes in the males, it was slightly but significantly elevated in the APOB and APOB/HSP females (167%, p < 0.05 and 161%, p < 0.05, respectively). APOB-100 overexpression alone did not influence *Fgf21* expression in the females. However, the *Fgf21* mRNA level was significantly higher in the APOB/HSP females when compared to the WT group (240%, p < 0.05). In contrast, in males, APOB-100 overexpression led to a decreased level of *Fgf21* expression (43%, p < 0.05), which was not influenced by human HSPB1 overexpression (45%, p = 0.083). The gene expression level of leptin (*Lep*) showed a more than twofold increase in APOB and APOB/HSP males compared to WT males (220%, p < 0.01; 248%, p < 0.01, respectively). In APOB females, however, we found a higher, almost fivefold increase (489%, p < 0.01) of *Lep* expression that was even higher in the APOB/HSP females (563%, p < 0.001). mRNA level of *Tnf* was remarkably elevated in response to APOB-100 overexpression in the vWAT of male animals (334%, p < 0.001), showing a slightly lower level in the APOB/HSP group (252%, p < 0.001). In contrast, *Tnf* expression failed to increase significantly either in APOB or APOB/HSP female groups. We found an increased level of transforming growth factor β1 (*Tgfb*) expression in APOB animals, both in females and males (188%, p < 0.01 and 235%, p < 0.001), which was not influenced by human HSPB1 overexpression. The expression level of Cd44 showed more than a fourfold increase in APOB groups in both males and females (410%, p < 0.01, and 472%, p < 0.01, respectively). Interestingly, human HSPB1 overexpression had a sex-dependent effect on *Cd44* expression. In APOB/HSP females, we found an even higher level of *Cd44* when compared to WT animals (683%, p < 0.001), while a decreasing expression level was detected in APOB/HSP males (256%, p < 0.05). Finally, the expression level of the gene encoding HSPB5 (*Cryab*) showed a significantly increased level in APOB and APOB/HSP animals compared to the WT animals, both in female and male groups.

### Non-alcoholic fatty liver disease develops only in HFD-fed APOB-100 males

**Fig. 4:**
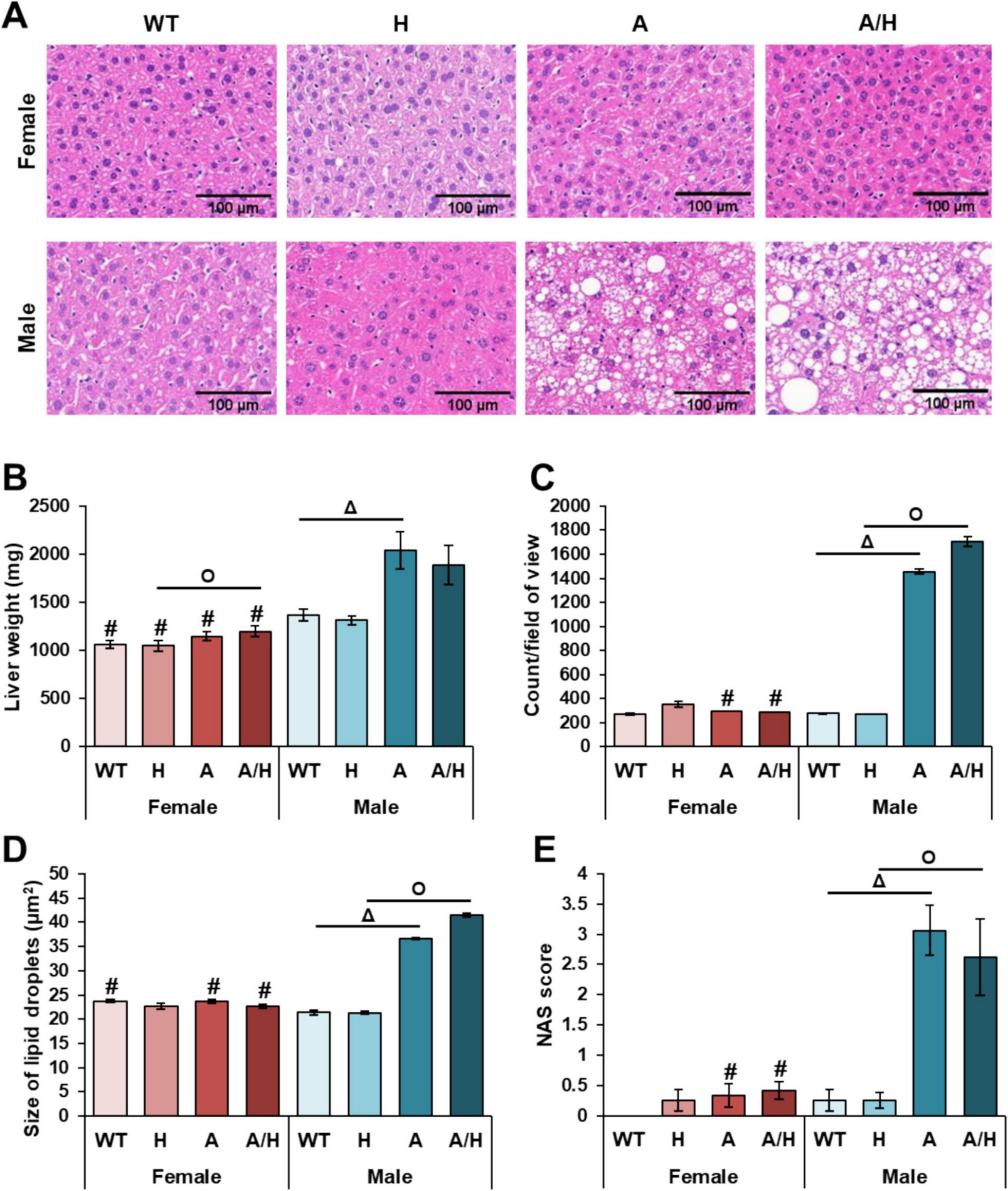
The effects of sex, MetS, and human HSPB1-overexpression on non-alcoholic fatty liver disease (NAFLD) at 10 months of age in female and male mice. Wild-type (WT) and HSPB1 (H) groups were on a standard diet. APOB-100 (A) and APOB-100/HSPB1 (A/H) MetS model mice were fed a high-fat diet. **A** Representative hematoxylin–eosin-stained sections, **B** liver weight, **C** number, and **D** size of lipid droplets, **E** NAFLD Activity Score (NAS). Values are mean ± SEM; n = 11–12; ^Δ^p < 0.05, A vs. WT; ^O^p < 0.05, A/H vs H; ^#^p < 0.05 Female vs. Male

At the end of the experiment, the livers were isolated and weighed, followed by hematoxylin–eosin staining of tissue sections to reveal pathological changes (Fig. 4A). In line with the body weight changes, the liver weight of the male APOB group was significantly higher compared to the male WT group (Fig. 4B). In contrast, the liver weight failed to increase in the APOB females despite their higher body weight compared to WT females. On the other hand, a slight but significant increase was found in the liver weight of APOB/HSP females compared to the sex-matched HSP group. Accordingly, hepatic lipid accumulation was more pronounced on hematoxylin–eosin-stained slides in male APOB animals compared to APOB females. Only a few small lipid droplets were found in the liver of the WT and HSP animals in both sexes and the APOB and APOB/HSP females. However, in the liver of APOB and APOB/HSP males, the number and size of lipid droplets were significantly higher compared to the male WT and HSP groups, respectively, and the corresponding female groups (Figs. 4C and D). Moreover, in the male APOB and APOB/HSP groups, the increasing levels of steatosis and hepatocyte ballooning led to significantly higher NAS as well (Fig. 4E). In contrast, no remarkable signs of inflammation were observed in either the APOB or APOB/HSP groups in both sexes. Human HSPB1 overexpression did not significantly affect liver pathology, as the level of NAS and the number and size of lipid droplets were only slightly different between the human HSPB1-overexpressing and the corresponding control groups.

### Increased hepatic Lpl expression was restored by human HSPB1 overexpression in APOB-100 females

**Fig. 5:**
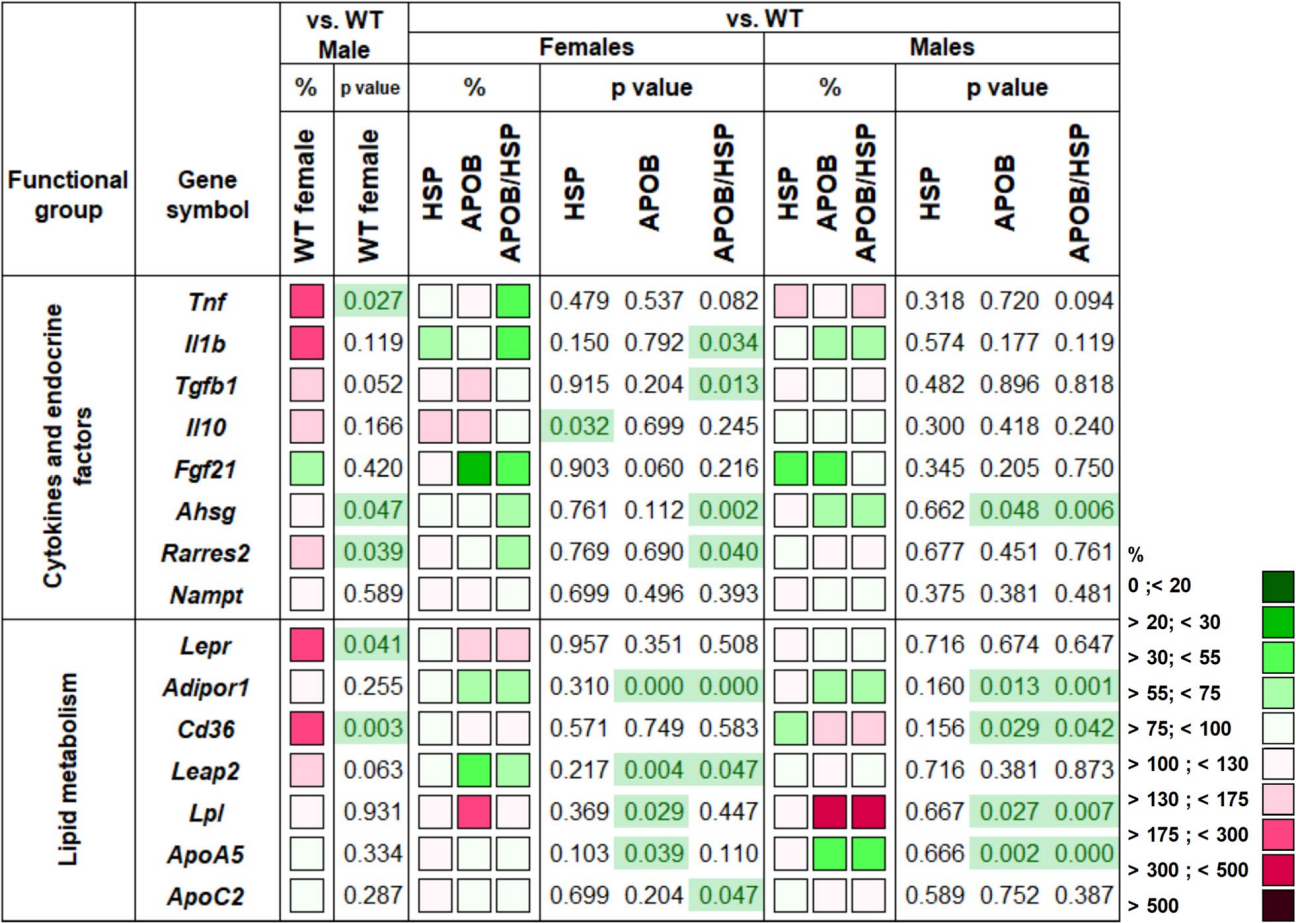
Heatmap of relative gene expression differences in the liver in response to sex, MetS, and HSPB1-overexpression. Relative expressions of several genes related to cytokines, endocrine factors, and lipid metabolism were studied in the liver of female and male mice using qPCR (n = 6). Wild-type (WT) and HSPB1 (HSP) groups were on a standard diet. APOB-100 (APOB) and APOB-100/HSPB1 (APOB/HSP) MetS model mice were fed a high-fat diet. For the WT female vs. WT male comparison, the relative expression of target genes in females was compared to the expression levels detected in males (results are given in percentages, where the male groups’ value = 100%). For HSP vs. WT, APOB vs. WT, and APOB/HSP vs. WT comparisons, the relative expression of target genes in transgenic animals was compared to the expression levels detected in WT female and male animals separately (results are given as a percentage, where WT groups’ value = 100%)

To reveal the molecular mechanism behind the pathological changes in the liver, the expression levels of genes involved in the regulation of inflammation, glucose, and lipid metabolism were analyzed by qPCR (Fig. 5). Performing a direct comparison of WT female vs. WT male groups we found significantly higher levels of *Tnf, Lepr* and *Cd36* mRNA in the liver of WT females (197%, p < 0.05; 215%, p < 0.05; 206%, p < 0.01, respectively).

In response to human HSPB1 overexpression, hepatic expression of *Il1b*, *Tgfb1*, α−2-HS-glycoprotein (*Ahsg*) and retinoic acid receptor responder (*Rarres2*) (43% p < 0.05, 79%, p < 0.05; 68%, p < 0.01;74%, p < 0.05 respectively) were significantly decreased and the expression of the further studied cytokines and hormone-like molecules tended to decrease in APOB/HSP females compared to the sex-matched WT group.

The expression level of the adiponectin receptor 1 (*Adipor1*) gene showed a slight (~ 70% compared to wild-type animals) but significant decrease in all APOB-100-overexpressing groups. The expression of liver-expressed antimicrobial peptide 2 (Leap-2) failed to change in the transgenic male groups. However, it was reduced in APOB and APOB/HSP females compared to the WT females (51%, p < 0.01 and 63%, p < 0.05, respectively). *Lpl* expression showed a remarkable increase in APOB and APOB/HSP males compared to the WT (337%, p < 0.05, and 415%, p < 0.01, respectively). On the other hand, we found a milder increase of *Lpl* expression in APOB females (263%, p < 0.05), which was restored by human HSPB1 overexpression (117%, p = 0.447). Finally, the apolipoprotein A-V (*Apoa5*) gene expression level only slightly decreased in the liver of APOB females. However, it was nearly halved in both APOB and APOB/HSP males (54%, p < 0.01 and 53%, p < 0.001, respectively).

### APOB-100 overexpression failed to increase the serum parameters of chronic kidney disease

**Fig. 6:**
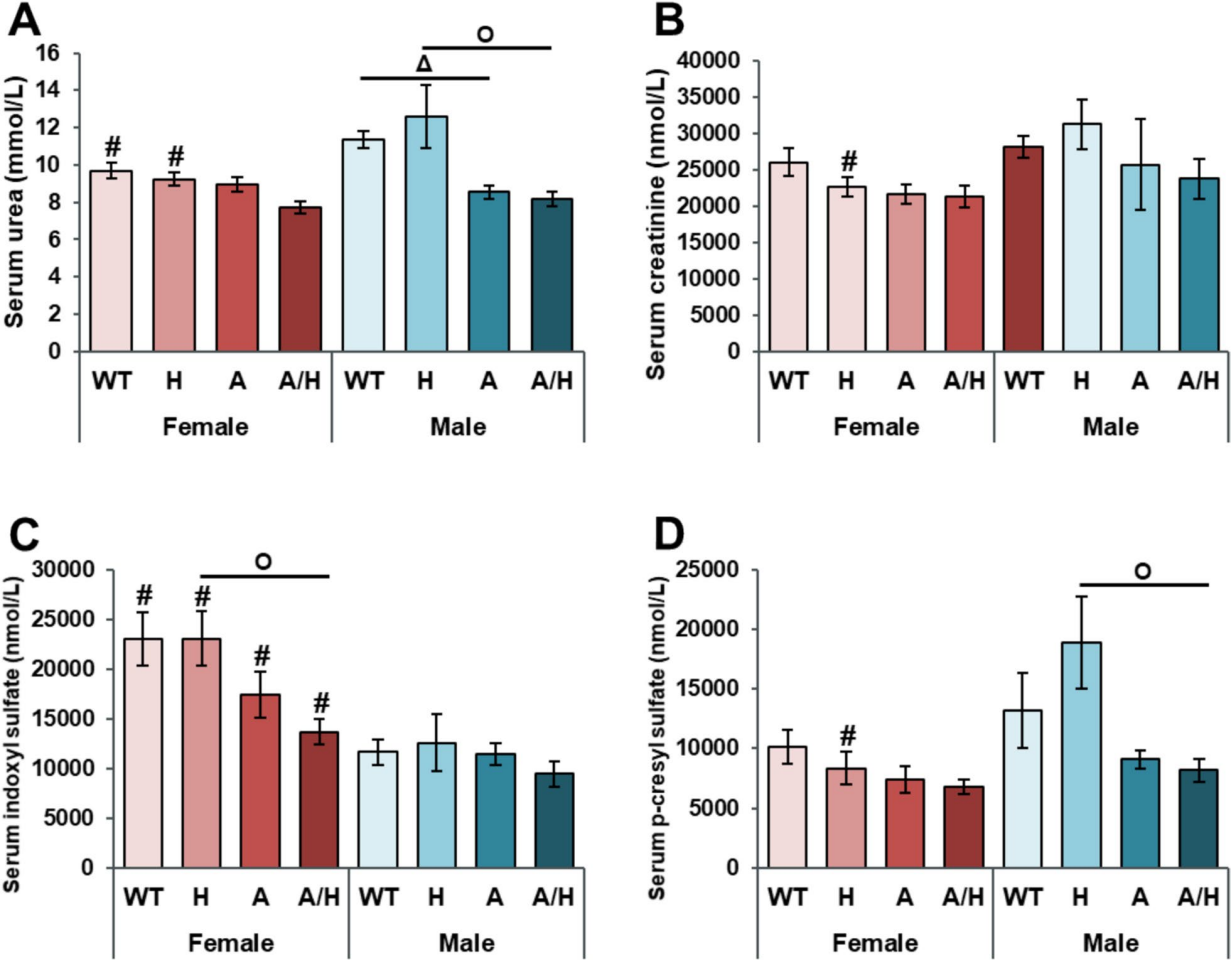
The effects of sex, MetS, and HSPB1-overexpression on the serum metabolite concentrations related to chronic kidney disease at 10 months in female and male mice. Wild-type (WT) and HSPB1 (H) groups were on a standard diet. APOB-100 (A)- and APOB-100/HSPB1 (A/H) MetS model mice were fed a high-fat diet. Serum **A** urea, **B** creatinine, **C** indoxyl sulfate, and **D** para-cresyl sulfate. Values are mean ± SEM; n = 9–12; ^Δ^p < 0.05, A vs. WT; ^O^p < 0.05, A/H vs H; ^#^p < 0.05 Female vs. Male

To investigate renal function, the serum levels of urea and creatinine and the uremic toxins indoxyl-sulfate and p-cresyl sulfate were measured. Interestingly, serum urea concentrations were significantly lower in the APOB and APOB/HSP males compared to the WT and HSP male groups, respectively (Fig. 6A). In females, the serum indoxyl sulfate levels tended to decrease in the APOB group compared to the WT group and significantly reduced in the APOB/HSP group compared to the HSP group (Fig. 6C). In males, the p-cresyl sulfate levels tended to decrease in the APOB group compared to the WT group and significantly reduced in the APOB/HSP group compared to the HSP group (Fig. 6D).

### Cardiac morphological and functional alterations developed in APOB-100 animals

**Fig. 7:**
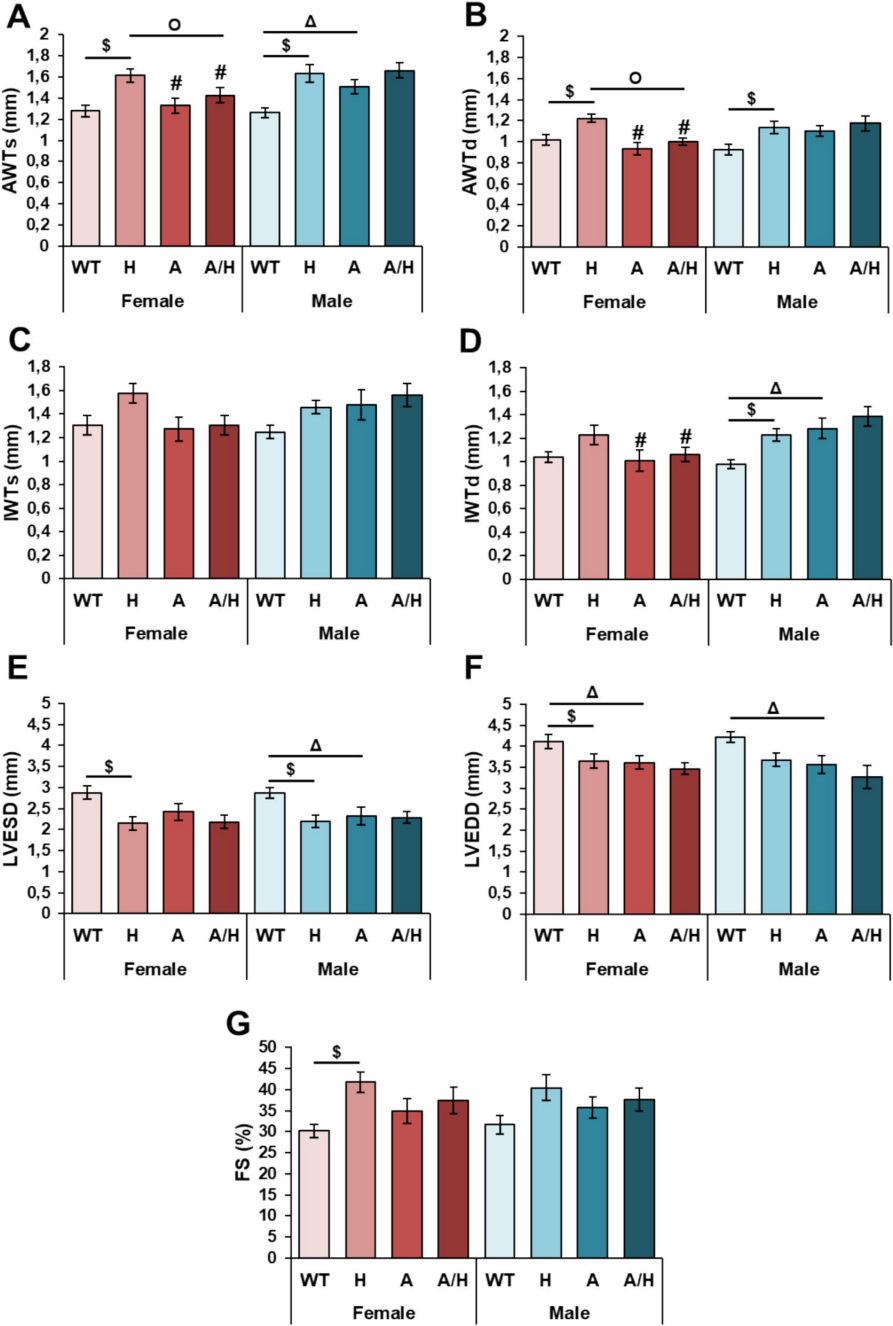
The effects of sex, MetS, and HSPB1-overexpression on cardiac performance at 9 months in female and male mice. Wild-type (WT) and HSPB1 (H) groups were on a standard diet. APOB-100 (**A**) and APOB-100/HSPB1 (A/H) MetS model mice were fed a high-fat diet. Anterior wall thicknesses in **A** systole and **B** diastole (AWTs and AWTd, respectively). Inferior wall thicknesses in **C** systole and **D** diastole (IWTs and IWTd, respectively). Left ventricular **E** end-systolic and **F** end-diastolic diameters (LVESD and LVEDD, respectively) and **G** fractional shortening (FS). Values are mean ± SEM; n = 11–12; ^$^p < 0.05, H vs. WT, ^Δ^p < 0.05, A vs. WT; ^O^p < 0.05, A/H vs H; ^#^p < 0.05 Female vs. Male

**Fig. 8:**
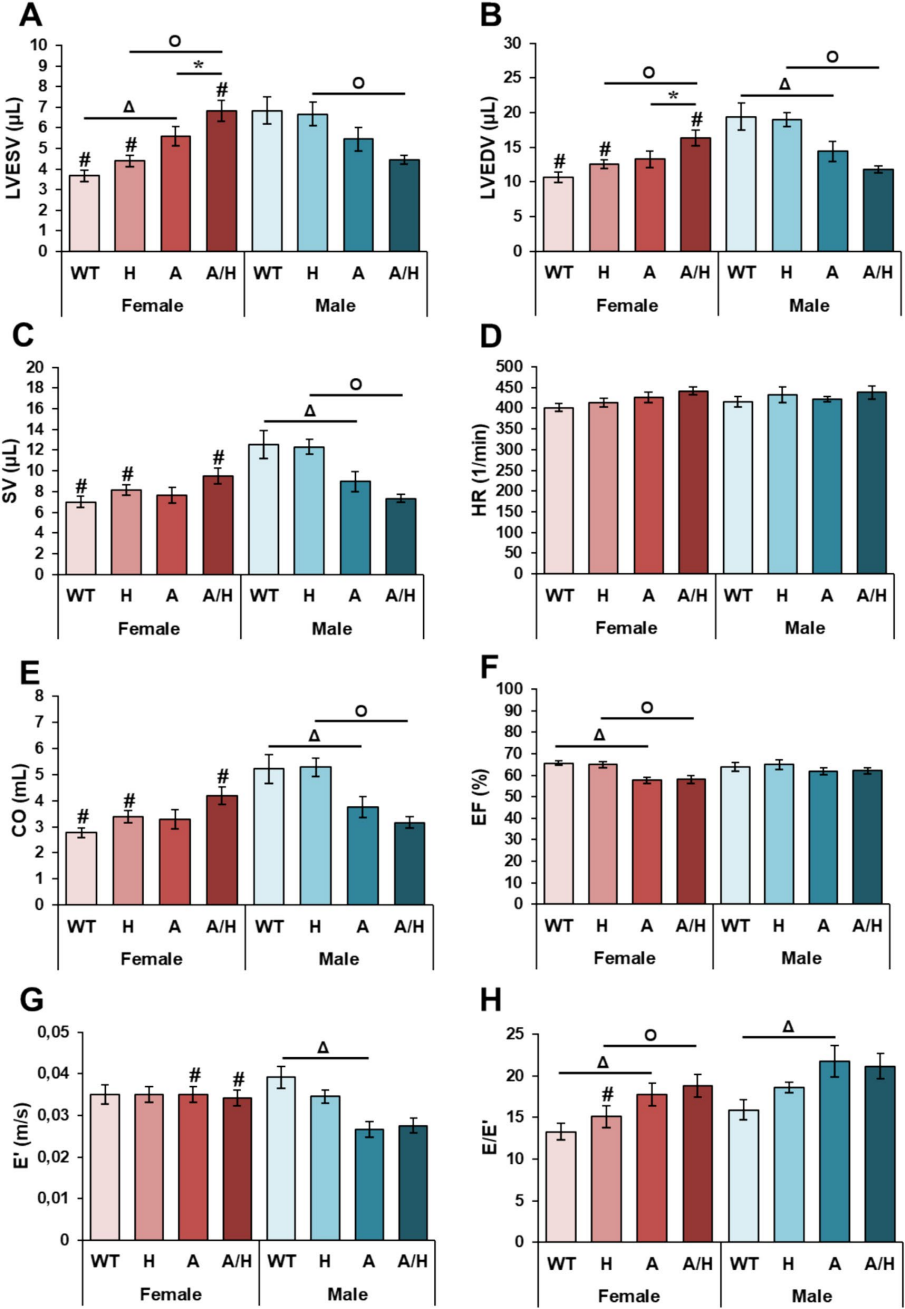
The effects of sex, MetS, and HSPB1-overexpression on echocardiographic parameters at 9 months in female and male mice. Wild-type (WT) and HSPB1 (H) groups were on a standard diet. APOB-100 (A) and APOB-100/HSPB1 (A/H) MetS model mice were fed a high-fat diet. **A** Left ventricular end-diastolic volume and **B** end-systolic volume (LVEDV and LVESV, respectively), **C** stroke volume (SV), **D** heart rate (HR), **E** cardiac output (CO), **F** ejection fraction (EF), **G** mitral annular e’ velocity (e’), and **H** mitral E/e’ ratio. Values are mean ± SEM; n = 11–12; ^$^p < 0.05, H vs. WT, ^Δ^p < 0.05, A vs. WT; ^O^p < 0.05, A/H vs H; ^#^p < 0.05 Female vs. Male

To reveal whether APOB-100 overexpression influences cardiac morphology and function, transthoracic echocardiography was performed at month 9 (Figs. 7A–G and 8A–H).

In the APOB females, there were no significant differences in the wall thicknesses compared to the wild-type females (Figs. 7A–D). However, the LVEDD was significantly lower in the APOB females compared to the WT females, suggesting an impaired relaxation (Fig. 8E). In contrast, the LVESV was markedly increased, suggesting impaired contraction, and the LVEDV showed an increasing tendency (p = 0.066), resulting in a reduced EF as compared to the wild-type females (Figs. 8A, B and F). Additionally, E/e’ was markedly higher due to the significantly elevated E velocity (0.62 ± 0.05 vs. 0.45 ± 0.03 ms, p < 0.05) in the APOB females compared to the WT females (Fig. 8H).

Interestingly, in the APOB males, the anterior wall thickness in systole (AWTs) and the inferior wall thickness in diastole (IWTd) were significantly thicker compared to WT males (Figs. 7A and D). Moreover, the LVESD and LVEDD were markedly decreased, and the FS tended to increase in the APOB males compared to WT males (Figs. 7E–G), suggesting the development of LVH. Indeed, the LVEDV, SV, and CO were markedly reduced in the APOB males as compared to the wild-type males (Figs. 8A, C and E). However, the EF failed to significantly reduce in the APOB males compared to the WT males (Fig. 8F). Moreover, the e’ was significantly decreased, and the E/e’ was markedly higher in the APOB males than in the sex-matched WT group, suggesting the development of diastolic dysfunction (Figs. 8E and F).

Interestingly, cardiomyocyte cross-sectional areas (Fig. S2A) were significantly lower in females compared to the group-matched males, irrespective of genotype, confirming the sex-based differences in heart size. There was no significant difference in the left ventricular collagen content among the groups (Fig. S2B).

### Human HSPB1 overexpression induced left ventricular hypertrophy without diastolic dysfunction in the wild-type mice

In the HSP groups, the systolic and diastolic anterior walls were markedly thicker, the systolic inferior wall thickness (IWTs) showed an increasing tendency (p = 0.091 in females), and the LVESD was significantly decreased compared to the sex-matched WT groups (Figs. 7A–E), suggesting the development of LVH. Moreover, in the HSP males, the IWTd was also significantly increased compared to the WT males (Fig. 7D). Indeed, the systolic parameter FS was increased in response to human HSPB1 overexpression compared to the sex-matched WT groups (Fig. 7G). Interestingly, there were no significant differences in the LVEDV, LVESV, SV, CO, EF, e’, and E/e’ between the HSP and WT groups (Figs. 8C–F).

### Human HSPB1 overexpression did not affect the APOB-100 overexpression-induced cardiac morphological alterations

There were no significant differences in the measured LV wall thicknesses and diameters, hemodynamic and diastolic functional parameters between the APOB/HSP and APOB animals (Figs. 7 and 8).

### Human HSPB1 overexpression increased the left ventricular Lepr expression in males, particularly in the APOB/HSP group

qPCR results revealed a significantly lower expression of *Tnf* and a significantly higher expression of *Lepr* genes in the left ventricle of WT females compared to males (Fig. S3). Furthermore, human HSPB1 overexpression led to elevated expression of *Lepr* in males, particularly in the APOB/HSP group (241%, p < 0.01).

## Discussion

In the present study, we analyzed the effects of human HSPB1 on obesity-related complications and comorbidities by crossing a human HSPB1-overexpressing strain with a mouse model of MetS overexpressing human APOB-100. Here, we confirm that several characteristics of MetS, including elevated serum glucose and TG levels, hepatic steatosis, and cardiac dysfunction, are more pronounced in male HFD-fed APOB-100-overexpressing animals compared to females [41, 42]. In addition, here we demonstrated that human HSPB1 overexpression also shows sex-dependent effects on weight gain, hypercholesterolemia, and hepatic and vWAT gene expression.

Although HSPs are traditionally considered intracellular proteins, their secretion is now a well-described phenomenon. Indeed, HSPB1 has been widely characterized as an internal protein cargo of secreted exosomes [48, 49]. Extracellular small molecular weight HSPs are also associated with different pathological conditions. HSPB1 has been detected in the serum of patients with several types of cancer, chronic pancreatitis, or acute ischemic stroke [50–52]. Additionally, myocardial cells were observed releasing HSPB1 after ischemia [53]. More interestingly, Rayner et al. reported that acylated LDL and estrogen treatment increased HSPB1 secretion. Accordingly, in their study, the serum level of HSPB1 was higher in HFD-fed APOE^−/−^ female mice compared to males, and it was inversely correlated with the area of atherosclerotic lesions. Moreover, extracellular HSPB1 reduced the specific uptake of acylated LDL by macrophages, thereby mitigating foam cell formation and concomitant inflammation and indicating the estrogen-dependent atheroprotective effect of circulating HSPB1 [48, 54]. On the other hand, human HSPB1 overexpression elicited a further body weight gain in the female HFD-fed APOB-100-overexpressing mice, while the body weight of males remained unaltered in our present study. Moreover, one of the most important observations of our present study is the opposite effect of human HSPB1 overexpression on serum LDL-cholesterol levels in the two sexes. Here, we detected an increasing trend in the LDL concentrations in the female APOB/HSP group and markedly lower serum LDL levels in the male APOB/HSP compared to their sex-matched controls. The decreased serum LDL concentration in the APOB/HSP males suggests a direct protective effect of human HSPB1 overexpression against hypercholesterolemia. On the other hand, HSPB1, similar to HSPB5 and HSPB2, may also be involved in the development of HFD-induced metabolic alterations based on the higher body weight and serum LDL concentration in the APOB/HSP females in our present study. To better understand how human HSPB1 influences the progression of MetS, we further investigated the underlying mechanisms of obesity-related complications, including inflammation, NAFLD, and impaired cardiac function and morphology.

In line with our previous results [41], qPCR data revealed a significantly lower *Fgf21* and *Tgfb1* and significantly higher *Lepr* expression levels in the vWAT of healthy WT females compared to males. In response to obesity, *Lep* expression was significantly elevated in both sexes; however, the rate of increase was higher in females. On the other hand, *Tnf* expression was significantly induced only in the obese male groups (i.e., APOB and APOB/HSP) and showed a slight decreasing tendency in response to human HSPB1 overexpression. Interestingly, despite the higher body weight of the obese female groups, *Tnf* expression changed neither in the APOB nor the APOB/HSP female group. These results suggest that human HSPB1 overexpression may suppress *Tnf* expression in the APOB animals in both sexes. The elevated secretion of TNF-α by adipose tissue can contribute to the development of many obesity-related complications, such as IR [55, 56], hypertriglyceridemia [57], NAFLD [58] or cardiac diseases [59]. Therefore, the lack of a significant increase in *Tnf* expression in the vWAT of either APOB or APOB/HSP females supports our present findings on the less severe complications of MetS in females. In a study by Kang et al., the expression level of the membrane receptor CD44 was found to be elevated in the liver and WAT in obesity, showing its important role in the development of several MetS-related complications, such as IR, steatosis, or inflammation [60]. In line with the literature, we found a significantly higher *Cd44* gene expression level in the vWAT of APOB animals in both sexes. While *Cd44* expression increased slightly in the APOB/HSP females, it nearly halved in APOB/HSP males. It has been reported that macrophage infiltration and *Tnf* expression were lower in the adipose tissue of HFD-fed CD44 KO mice compared to HFD-fed WT mice [60, 61]. Indeed, in our present study, the reduced *Cd44* expression in our HFD-fed APOB male mice further supports the inflammation-mitigating effects of human HSPB1 overexpression. Although this hypothesis seems to be contradicted by the elevated *Cd44* in APOB/HSP females, a recent study revealed that CD44 inactivation in female mice had no detectable effects on several obesity-related symptoms, including adipose tissue inflammation [61].

In our present study, another interesting sex-dependent effect of obesity and human HSPB1 overexpression was observed for *Fgf21,* a peptide hormone involved in energy homeostasis regulation. *Fgf21* was significantly repressed in the APOB male group and was not influenced by human HSPB1 overexpression in vWAT. However, *Fgf21* mRNA level was slightly increased in APOB females and was significantly elevated in the APOB/HSP group. Although the liver is the primary source of circulating FGF21, it is also expressed by other organs, such as adipose tissues [62]. Moreover, previous studies suggested that FGF21 might play an autocrine role in several adipocyte functions, such as glucose uptake [63] or browning of the WAT [64]. In HFD-fed IR mice, WAT-specific overexpression of *Fgf21* improved metabolic function by reducing inflammation, IR, and hepatic steatosis [65]. We hypothesize that the high expression level of *Fgf21* in the vWAT of APOB/HSP females may contribute to maintaining metabolic health despite their higher body weight.

Similar to our previous results [41], the development of NAFLD was detected only in male APOB animals in response to HFD. However, neither the weight of the liver nor lipid droplet accumulation was increased in female APOB mice. In addition to the effects of estrogen [66], the higher leptin sensitivity in female mice may also contribute to this phenomenon. This hypothesis is supported by the higher hepatic *Lepr* expression in females detected in our current and previous experiments [41]. In addition, the expression levels of *Leap2* and *Apoa5* showed sex-specific changes in APOB mice. Previous studies have demonstrated that serum levels of the peptide hormone LEAP2 are markedly elevated in patients with metabolic dysfunction-associated steatohepatitis, whereas only moderate increase was observed in simple steatosis. Despite lipid accumulation, no signs of inflammation were detected in the liver of our APOB male mice, suggesting that these animals may be in the early, reversible stage of disease progression [67], consistent with the modest increase in *Leap2* expression. In contrast, APOB females exhibited a surprising reduction in *Leap2* expression, which may contribute to their resistance to steatosis [68]. Having an important role in triglyceride metabolism, APOA5 has also been implicated in the development of NAFLD; however, results remain controversial. A recent study showed that Apoa5 knockout hamsters develop hepatic steatosis even on normal chow diet [69]. In line with this, APOB males in our study showed a more pronounced downregulation of *Apoa5* expression compared to females, further supporting the observed histopathological sex differences.

Moreover, here, we found that the expression level of *Lpl* was elevated in the liver of APOB mice in both sexes, with a greater increase observed in males. Notably, while male APOB mice showed a further increase in *Lpl* expression in response to human HSPB1 overexpression, it was normalized in APOB/HSP females. *Lpl* is an important lipid metabolism regulator responsible for the hydrolysis of TGs in lipoproteins. Although its expression level is typically low in the adult liver, it was found to be increased in NAFLD patients [70, 71]. Additionally, liver-specific overexpression of *Lpl* accelerated hepatic TG accumulation [72], suggesting a pathophysiological role of LPL in the development of NAFLD. Accordingly, we observed the highest expression level of *Lpl* in the liver of APOB and APOB/HSP males, which was parallel with the characteristic signs of hepatic steatosis. On the other hand, the *Lpl* expression was also increased in APOB females without NAFLD, suggesting that factors other than *Lpl* are also necessary for developing NAFLD. Nevertheless, the normalized *Lpl* expression in the APOB/HSP females may contribute to the absence of hepatic lipid accumulation despite their higher body weight and serum LDL level. The significant increase of NAS in the liver of APOB and APOB/HSP males was due to the massive steatosis and, to a lesser extent, ballooning degeneration of the hepatocytes. However, we failed to observe remarkable signs of inflammation, suggesting a relatively early stage of the disease [67]. Indeed, none of the studied cytokines showed increased hepatic expression in obese APOB and APOB/HSP animals. In contrast, we observed a decreasing trend in the hepatic expression level of the pro-inflammatory genes in APOB/HSP females.

Obesity is also known to increase the risk of chronic kidney disease both directly and indirectly via systemic inflammation and comorbidities, such as diabetes mellitus or hypertension [73]. Therefore, our next objective was to determine whether chronic kidney disease develops in our HFD-fed APOB model. None of the investigated serum parameters were increased in APOB or APOB/HSP animals, suggesting that renal function is not impaired in our models. Contrary to our expectations, the chronic kidney disease-related serum parameters tended to decrease in the obese APOB and APOB/HSP animals, probably due to inadequate protein intake and reduced muscle mass associated with HFD. Indeed, HFD feeding impaired muscle protein synthesis in aged animals. Therefore, it may accelerate the age-related loss of muscle mass and function, contributing to sarcopenic obesity [74, 75]. Notably, several metabolites associated with renal dysfunction showed a reduced serum level in sarcopenia in the study of Kameda et al. [76].

In our present study, in APOB males, the LVH and diastolic dysfunction were accompanied by preserved EF, probably due to proportionally reduced LVEDV and LVESV, decreased SV, and CO. In contrast, APOB females showed disproportionally increased left ventricular volumes and consequently reduced EF with mild diastolic dysfunction. Although previous studies suggested that HSPs have cardioprotective roles [77], human HSPB1 overexpression failed to ameliorate the cardiac alterations in our APOB model. Unexpectedly, male and female human HSPB1-overexpressing WT animals developed LVH without diastolic dysfunction. Interestingly, it has been shown that exercise training increases the expression of several HSPs, including HSPB1, in cardiac tissue. In contrast, reduced expression of the heat shock transcription factor (HSF1) impaired the adaptive response to exercise and led to cardiac dysfunction, suggesting an important role of HSPs in the development of exercise-induced physiological hypertrophy [78]. Our present results raise the possibility that human HSPB1 overexpression may not only inhibit hypertrophy-associated cardiac dysfunction but also be involved in the development of adaptive hypertrophy.

### Perspectives and significance

In conclusion, human HSPB1 may be involved in the regulation of obesity-related metabolic alterations; however, it has sex-dependent effects. At first sight, the increased weight gain and serum LDL level in the APOB/HSP females suggest that the overexpression of human HSPB1 contributes to the detrimental consequences of obesity. However, despite the higher body weight of the APOB/HSP females, none of the investigated obesity-related complications and comorbidities, such as inflammation, hepatic steatosis, or cardiac dysfunctions, worsened in them. Therefore, human HSPB1 overexpression may compensate for the detrimental effects of HFD in APOB females, enabling them to increase their energy sources without negative consequences. Based on the estrogen-dependent atheroprotective function of extracellular human HSPB1 in previous studies, human HSPB1 may have a complex regulatory role in obesity-related comorbidities. A more profound understanding of these mechanisms can potentially facilitate the development of therapeutic applications in the future. Nevertheless, it is important to take into account sex-based differences to ensure optimal therapeutic outcomes.

## Supplementary Information

Additional file 1

## Data Availability

The datasets used and/or analyzed during the current study are available from the corresponding author on a reasonable request.

## Abbreviations

*Adipor1*: Adiponectin receptor 1
*Ahsg*: α-2-HS-glycoprotein
*Apoa5*: Apolipoprotein A-V
APOB-100: Apolipoprotein B-100
A-velocity: Atrial flow velocity
AWTd: Anterior wall thickness in diastole
AWTs: Anterior wall thickness in systole
CD44/*Cd44*: Hyaluronate receptor
CO: Cardiac output
*Cryab*: AB-crystallin
e’-velocity: Mitral annular velocity
EF: Ejection fraction
E-velocity: Early mitral flow velocity
FGF21/*Fgf21*: Fibroblast growth factor 21
FS: Fractional shortening
*Gapdh*: Glyceraldehyde-3-phosphate dehydrogenase
HDL: High-density lipoprotein
HFD: High-fat diet
HR: Heart rate
HSF1: Heat shock transcription factor 1
HSP: Heat shock protein
HSPB1: Heat shock protein family B member 1
IL-1b: Interleukin-1 β
IL-6: Interleukin-6
IR: Insulin resistance
IVRT: Isovolumic relaxation time
IWTd: Inferior wall thickness in diastole
IWTs: Inferior wall thickness in systole
LDL: Low-density lipoprotein
*Leap2*: Liver-expressed antimicrobial peptide 2
*Lep*: Leptin
*Lepr*: Leptin receptor
LPL/*Lpl*: Lipoprotein lipase
LVEDD: Left ventricular end-diastolic diameter
LVEDV: Left ventricular end-diastolic volume
LVESD: Left ventricular end-systolic diameter
LVESV: Left ventricular end-systolic volume
LVH: Left ventricular hypertrophy
MetS: Metabolic syndrome
NAFLD: Non-alcoholic fatty liver disease
PBS: Phosphate-buffered saline
PSFG: Picrosirius red and fast green
qPCR: Quantitative real-time polymerase chain reaction
*Rarres2*: Retinoic acid receptor responder (tazarotene induced) 2
RGB: Red–green–blue
SD: Standard diet
SV: Stroke volume
TG: Triglyceride
*Tgfb1*: Transforming growth factor β1
TNFα/*Tnf*: Tumor necrosis factor-α
vWAT: Visceral white adipose tissue
WT: Wild type
