# Supplementary material for: Overexpression of the human heat shock protein B1 alters obesity-related metabolic changes in a sex-dependent manner in a mouse model of metabolic syndrome
Source: Biol Sex Differ. 2025 Aug 25;16:65. doi: 10.1186/s13293-025-00746-z (PMC12379348; doi:10.1186/s13293-025-00746-z)
Supplement: Supplementary file 1 — Additional file 1 [file 13293_2025_746_MOESM1_ESM.docx]

**Supplementary material to:**

**Overexpression of the human heat shock protein B1 alters obesity-related metabolic changes in a sex-dependent manner in a mouse model of metabolic syndrome**

Zsófia Ruppert^1,2^, Márta Sárközy^3,4^, Bettina Rákóczi^1,2^, Brigitta Dukay^1^, Petra Hajdu^1^, Gergő Szűcs^4^, Zsolt Galla^5^, Ákos Hunya^1^, Ferenc Kovács^6,7^, András Kriston^6,7^, Péter Monostori^5^, Péter Horváth^6,7,8^, Gábor Cserni^9^, László Tiszlavicz^9^, Tamás Csont^4^, László Vígh^1^, Miklós Sántha^1^, Zsolt Török^1†^ and Melinda E. Tóth^1†^,

^1^Laboratory of Molecular Stress Biology, Institute of Biochemistry, HUN-REN Biological Research Centre, Szeged, Hungary

^2^Faculty of Science and Informatics, Doctoral School in Biology, University of Szeged, Szeged, Hungary

^3^Department of Pathophysiology, Albert Szent-Györgyi Medical School, University of Szeged, Szeged, Hungary

^4^MEDICS Research Group, Department of Biochemistry, Albert Szent-Györgyi Medical School, University of Szeged, Szeged, Hungary

^5^Metabolic and Newborn Screening Laboratory, Department of Pediatrics, Albert Szent-Györgyi Medical School, University of Szeged, Szeged, Hungary

^6^Synthetic and Systems Biology Unit, Institute of Biochemistry, HUN-REN Biological Research Centre, Szeged, Hungary

^7^Single-Cell Technologies Ltd, Szeged, Hungary

^8^Institute of AI for Health, Helmholtz Zentrum München, Neuherberg, Germany

^9^Department of Pathology, Albert Szent-Györgyi Medical School, University of Szeged, Szeged, Hungary

^†^These authors contributed equally to the work.


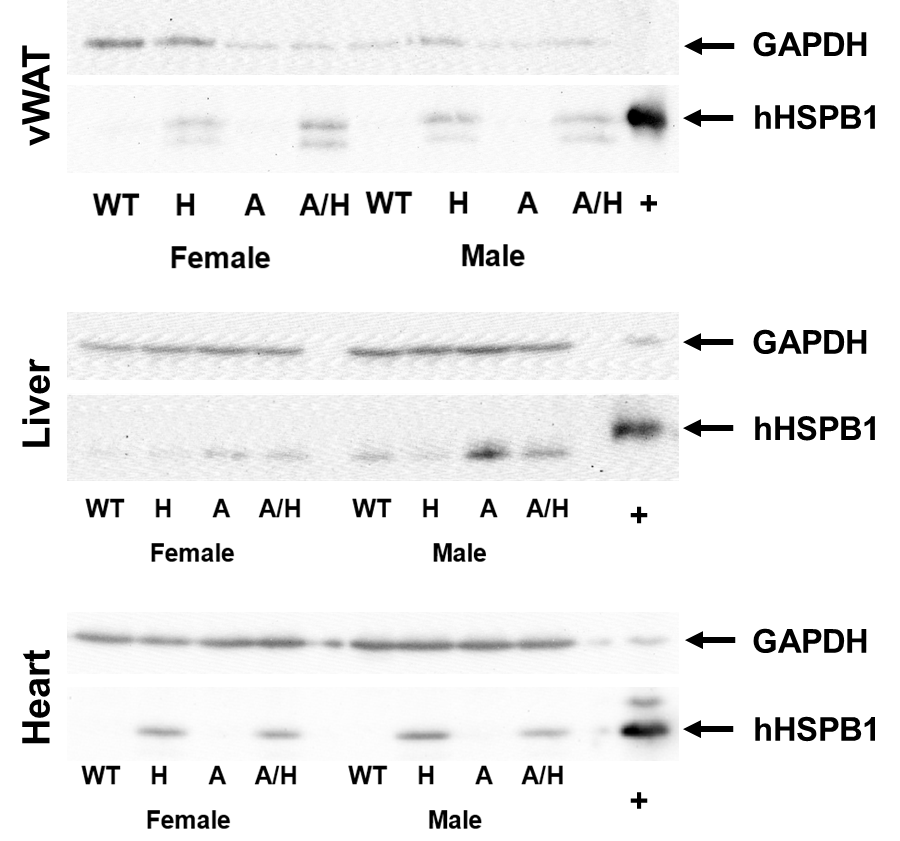


**Figure S1** Western blot analysis of transgenic human HSPB1 in the visceral white adipose tissue (vWAT), liver and the heart of female and male mice. Wild-type (WT) and HSPB1 (H) groups were on a standard diet. APOB-100 (A) and APOB-100/HSPB1 (A/H) MetS model mice were fed a high-fat diet.

***
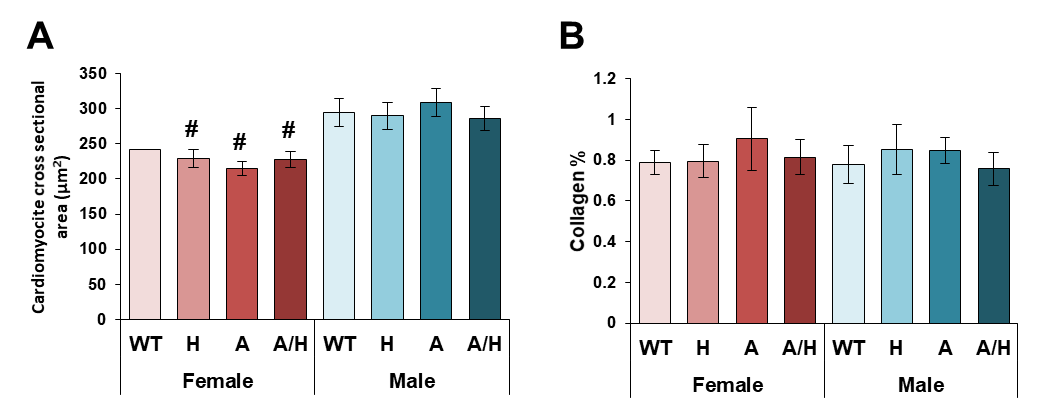
***

**Figure S2** The effects of sex, MetS, and HSPB1-overexpression on **A)** cardiomyocyte cross-sectional areas and **B)** left ventricular collagen content at 10 months in female and male mice. Wild-type (WT) and HSPB1 (H) groups were on a standard diet. APOB-100 (A) and APOB-100/HSPB1 (A/H) MetS model mice were fed a high-fat diet. Values are mean±SEM; n=11–12 for cardiomyocyte cross-sectional areas and n=3-12 for left ventricular collagen content; #p < 0.05 Female vs. Male.


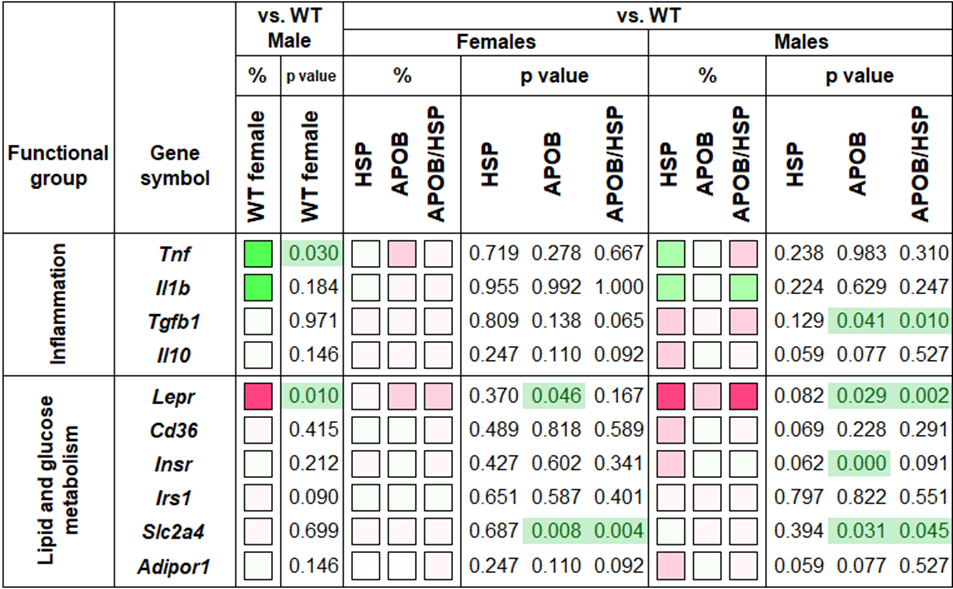

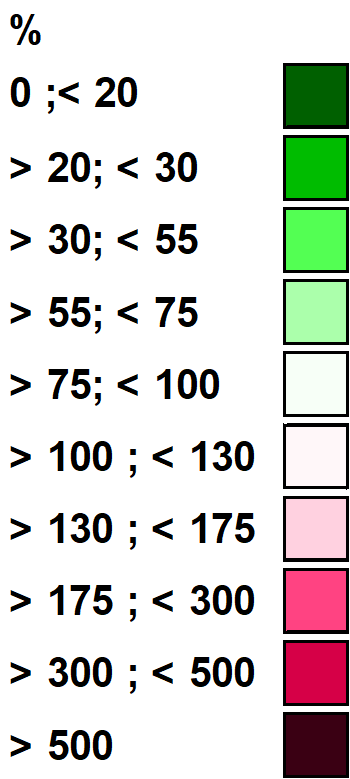


**Figure S3** Heatmap of relative gene expression differences in the heart in response to sex, MetS, and HSPB1-overexpression. Relative expressions of several genes related to inflammation, lipid, and glucose metabolism were studied in female and male mice using qPCR (n=6). Wild-type (WT) and HSPB1 (HSP) groups were on a standard diet. APOB-100 (APOB) and APOB-100/HSPB1 (APOB/HSP) MetS model mice were fed a high-fat diet. For the WT female vs. WT male comparison, the relative expression of target genes in females was compared to the expression levels detected in males (results are given in percentages, where the male groups’ value = 100%). For HSP vs. WT, APOB vs. WT, and APOB/HSP vs. WT comparisons, the relative expression of target genes in transgenic animals was compared to the expression levels detected in WT female and male animals separately (results are given as a percentage, where WT groups’ value = 100%).

**Table S1** Diet composition. High-fat Diet contains 0.15% of supplementary cholesterol (giving a total of 2% cholesterol).

**Table S2.** Primer sequences used for qPCR analysis.
